# Doxorubicin upregulates CXCR4 via miR-200c/ZEB1-dependent mechanism in human cardiac mesenchymal progenitor cells

**DOI:** 10.1038/cddis.2017.409

**Published:** 2017-08-24

**Authors:** Sara Beji, Giuseppina Milano, Alessandro Scopece, Lucia Cicchillitti, Chiara Cencioni, Mario Picozza, Yuri D'Alessandra, Sarah Pizzolato, Matteo Bertolotti, Gabriella Spaltro, Angela Raucci, Giulia Piaggio, Giulio Pompilio, Maurizio C Capogrossi, Daniele Avitabile, Alessandra Magenta, Elisa Gambini

**Affiliations:** 1 Vascular Pathology Laboratory, Istituto Dermopatico dell’Immacolata, IRCCS, Via dei Monti di Creta 104, Rome 00167, Italy; 2 Unit of Vascular Biology and Regenerative Medicine, Centro Cardiologico Monzino, IRCCS, Via Carlo Parea 4, Milan 20138, Italy; 3 Laboratory of Cardiovascular Research, Department of Surgery and Anesthesiology, University Hospital Lausanne; Rue du Bugnon 46, Lausanne 1011, Switzerland; 4 Department of Research, Advanced Diagnostics and Technological Innovation, Regina Elena National Cancer Institute, Via Elio Chianesi 53, Rome 00144, Italy; 5 Division of Cardiovascular Epigenetics, Department of Cardiology, Goethe University, Theodor-Stern-Kai 7, Frankfurt am Main 60590, Germany; 6 National Research Council (CNR), Institute of Cell Biology and Neurobiology, Via del Fosso di Fiorano, 64, Rome 00143, Italy; 7 Immunology and Functional Genomics Unit, Centro Cardiologico Monzino (CCM), IRCCS, Via Carlo Parea 4, Milan 20138, Italy; 8 Unit of Experimental Cardio-Oncology and Cardiovascular Aging, Centro Cardiologico Monzino (CCM), IRCCS, Via Carlo Parea 4, Milan 20138, Italy; 9 Department of Clinical Sciences and Community Health, University of Milan, Via Festa del Perdono 7, Milan 20122, Italy

## Abstract

Doxorubicin (DOXO) treatment is limited by its cardiotoxicity, since it causes cardiac-progenitor-cell depletion. Although the cardioprotective role of the stromal cell-derived factor-1/C-X-C chemokine receptor type 4 (SDF1/CXCR4) axis is well established, its involvement during DOXO-induced cardiotoxicity has never been investigated. We showed that in a mouse model of DOXO-induced cardiomyopathy, CXCR4^+^ cells were increased in response to DOXO, mainly in human cardiac mesenchymal progenitor cells (CmPC), a subpopulation with regenerative potential. Our *in vitro* results showed a CXCR4 induction after 24 h of DOXO exposure in CmPC. SDF1 administration protected from DOXO-induced cell death and promoted CmPC migration. CXCR4 promoter analysis revealed zinc finger E-box binding homeobox 1 (ZEB1) binding sites. Upon DOXO treatment, ZEB1 binding decreased and RNA-polymerase-II increased, suggesting a DOXO-mediated transcriptional increase in CXCR4. Indeed, DOXO induced the upregulation of miR-200c, that directly targets ZEB1. SDF1 administration in DOXO-treated mice partially reverted the adverse remodeling, decreasing left ventricular (LV) end diastolic volume, LV ejection fraction and LV anterior wall thickness in diastole, recovering LV end systolic pressure and reducing±d*P*/d*t*. Moreover, *in vivo* administration of SDF1 partially reverted DOXO-induced miR-200c and p53 protein upregulation in mouse hearts. In addition, downmodulation of ZEB1 mRNA and protein by DOXO was significantly increased by SDF1. In keeping, p21 mRNA, that is induced by p53 and inhibited by ZEB1, is induced by DOXO treatment and is decreased by SDF1 administration. This study showed new players of the DOXO-induced cardiotoxicity, that can be exploited to ameliorate DOXO-associated cardiomyopathy.

Anthracyclines are effective chemotherapeutic agents. Among them, Doxorubicin (DOXO) is largely used in different types of tumors, including breast cancer, esophageal carcinoma, osteosarcoma, sarcomas and lymphomas.^1^ Unfortunately, the clinical application of DOXO is limited by cumulative dose-dependent cardiotoxicity.^1^ In particular, DOXO-induced cardiotoxicity determines progressive cardiac dilation, contractile dysfunction and ultimately congestive heart failure.^2^ Studies in experimental animal models and human endomyocardial biopsies evidenced histological alterations associated to DOXO-induced cardiomyopathy, consisting of multiple areas of interstitial fibrosis that replace apoptotic and necrotic cardiomyocytes.^2, 3^ Oxidative stress and DNA damage are considered the key mechanisms involved in DOXO-mediated cardiotoxicity.^4, 5^

Although cardiomyocytes have been considered the most representative cellular targets, other cells, including endothelial cells (EC)^6^ and progenitor cells, are involved in DOXO-induced cardiomyopathy.^7, 8^ Indeed, DOXO, similarly to other anticancer drugs, such as Trastuzumab and Sorafenib, has been demonstrated to affect the survival and function of cardiac mesenchymal progenitor cells (CmPC), leading to a progressive loss of cardiac tissue homeostasis and eventually to congestive heart failure.^9, 10, 11, 12, 13^ The stromal cell-derived factor-1/C-X-C chemokine receptor type 4 (SDF1/CXCR4) axis is involved in many pathological conditions of tissue injury and stress, including cardiovascular diseases and myocardial infarction. After an ischemic insult, SDF1 acts as a chemoattractant to stimulate the homing of circulating CXCR4-positive cells, as well as of other stem cells, to the site of injury, for tissue regeneration and repair. In particular, SDF1 provides trophic support for cells, stimulates progenitor cell differentiation and promotes angiogenesis through a paracrine mechanism.^14^ Indeed, the activation of the SDF1/CXCR4 axis promotes extensive mobilization of CmPC and supports cardiac repair of the infarcted heart.^15, 16, 17^ Notably, the cardiac protective role of this axis has been recently confirmed in a clinical setting of ischemic heart failure.^18^ Moreover, in dilated cardiomyopathy, SDF1 increases and enhances the number of circulating progenitor cells^19^ and DOXO-induced cardiomyopathy promotes mesenchymal stem cell migration to the heart, where SDF1 expression is elevated.^20^

MicroRNAs (miRNAs) are 21–23 nucleotides RNA molecules that regulate the stability or translational efficiency of target messenger RNAs.^21^ miRNAs control a wide range of cell functions and have been associated with inflammation, oxidative stress and different pathologies, including heart failure, cardiac hypertrophy and myocardial arrhythmias.^22, 23^ Indeed, our group demonstrated that the entire miR-200 family is upregulated in endothelial cells upon oxidative stress.^24^ In particular, we demonstrated that miR-200c is the most upregulated family member in EC upon exposure to oxidative stress and that its increase is responsible for apoptosis and senescence via the inhibition of miR-200 family target zinc finger E-box binding homeobox 1 (ZEB1).^24^

In this paper, we showed that DOXO induces the *in vitro* and *in vivo* upregulation of CXCR4, making human CmPC more prone to respond to SDF1 stimulation. Moreover, we demonstrated that DOXO-induced CXCR4 upregulation in CmPC is mediated, at least in part, by a miR-200c/ZEB1 pathway. As a consequence, the activation of SDF1/CXCR4 axis promotes CmPC migration and improves cell survival upon DOXO treatment. Finally, the activation of the SDF1/CXCR4 axis ameliorates cardiac functional deficits in mice treated with cardiotoxic doses of DOXO via a miR-200c/ ZEB1/p53 pathway modulation.

## Results

### Doxorubicin increases CXCR4 expression *in vivo* and *in vitro*

**Figure 1:**
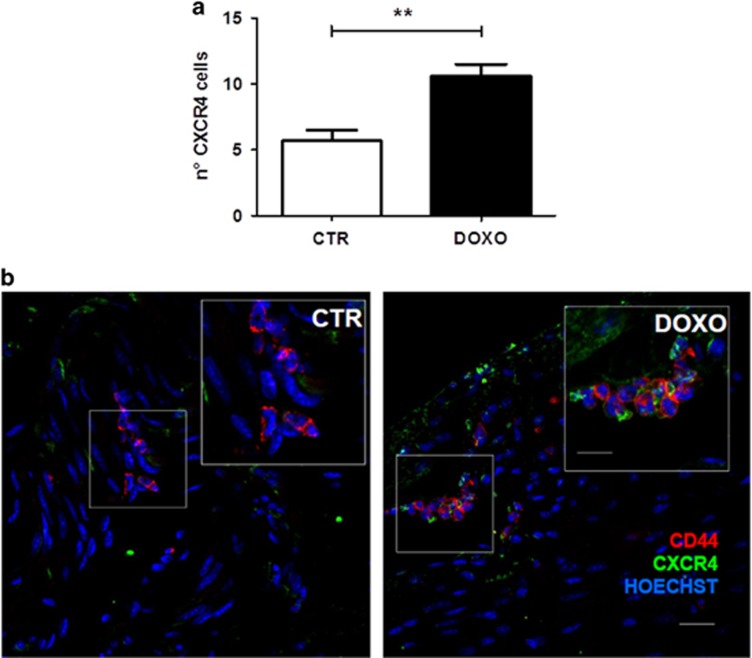
CXCR4 is increased *in vivo* in response to DOXO. (**a**) CXCR4^+^ cell quantitative analysis. The number of CXCR4^+^ cells from 9 randomly selected fields was counted for each tissue section of DOXO and untreated mice (CTR). The CXCR4^+^ cells were calculated as a percentage on the total number of nuclei in the same fields (9 different fields per mouse). The number of CXCR4^+^ cells were significantly higher in DOXO compared to CTR mice (**P<0.005). Data were representative of five independent experiments. Results are presented as mean±S.E.M. (**b**) Representative images of heart sections of CTR and DOXO mice, immunostained with CXCR4 (green) and CD44 (red) antibody. Nuclei are stained with Hoechst 33258 (blue). Scale bar: 20 *μ*m and 50 *μ*m in the inset

To dissect the mechanism by which SDF1 exerts its cardioprotective properties, the expression levels of the most characterized receptor CXCR4 was assessed in heart sections of DOXO-treated and untreated mice. Interestingly, the total number of CXCR4^+^ cells was significantly increased in response to 21 days of DOXO treatment (Figure 1a and Supplementary Fig S1a). Immunofluorescence analysis revealed that CXCR4 receptor was mainly upregulated in cells localized in the interstitial space between cardiomyocytes and CmPC, identified by the expression of the mesenchymal markers CD44 and CD29 (Figure 1b,Supplementary Fig S1b).^25, 26^ To further confirm these results *in vitro*, we characterized by flow cytometry CmPC treated or not with 1 *μ*M DOXO for 24 h (h). Results revealed that CmPC fully express CD44, CD29, CD73 and CD166 surface markers, confirming their mesenchymal cell origin. Moreover, we confirmed that after DOXO treatment the percentage of CXCR4^+^ cells increased (Supplementary Fig S2).

**Figure 2:**
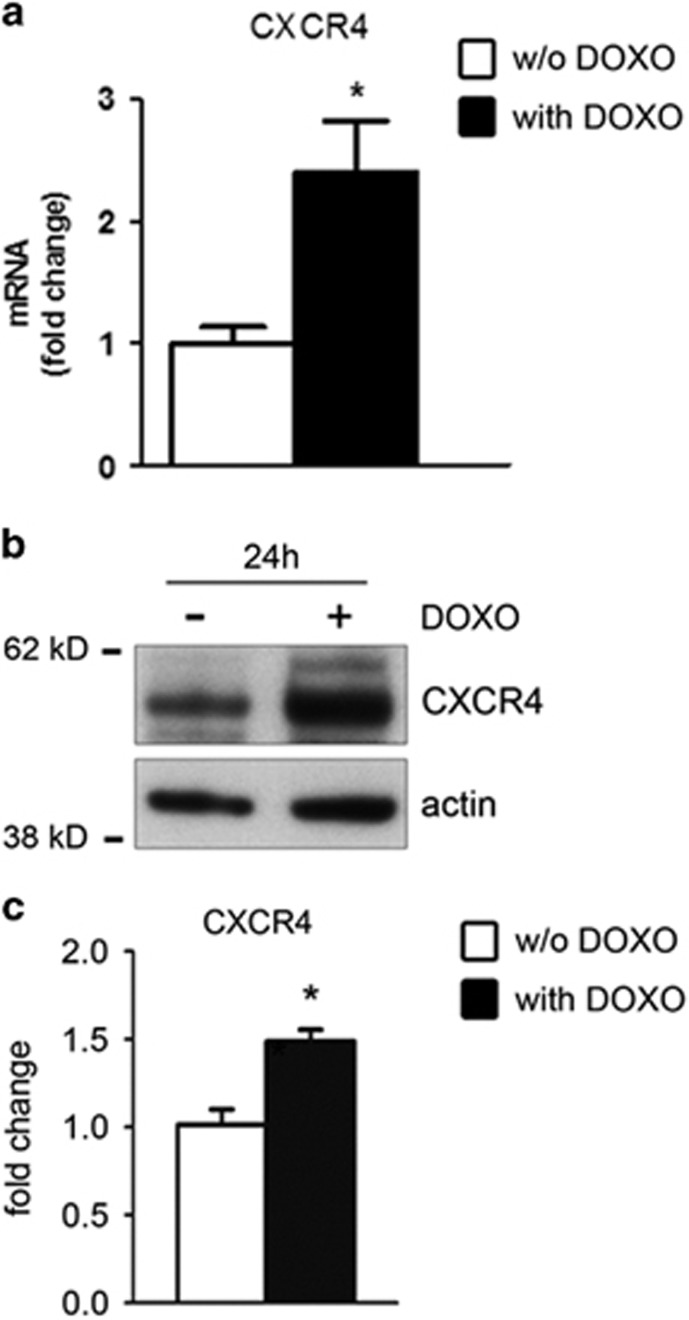
CXCR4 is induced in response to DOXO in CmPC. (**a**) qPCR analysis showing CXCR4 upregulation in CmPC treated with 1 *μ*M DOXO for 24 h as compared to untreated cells (*n*=3, *P<0.05) Results are presented as mean±S.D.; (**b**) Representative western blot showing an increase in CXCR4 expression in CmPC upon DOXO treatment. *β*-actin was used as a loading control. Actin indicate *β*-actin; (**c**) Expression levels of CXCR4 protein were evaluated by densitometric analysis and normalized by *α*-tubulin protein levels (*n*=3, *P<0.05)

To assess the effect of DOXO in human CmPC, CXCR4 expression was measured in CmPC treated with 1 *μ*M DOXO for 24 h. Interestingly, both the mRNA (Figure 2a) and protein levels of CXCR4 were significantly upregulated (Figures 2b and c).

### SDF1 induces migration and protects DOXO-treated CmPC from apoptosis

**Figure 3:**
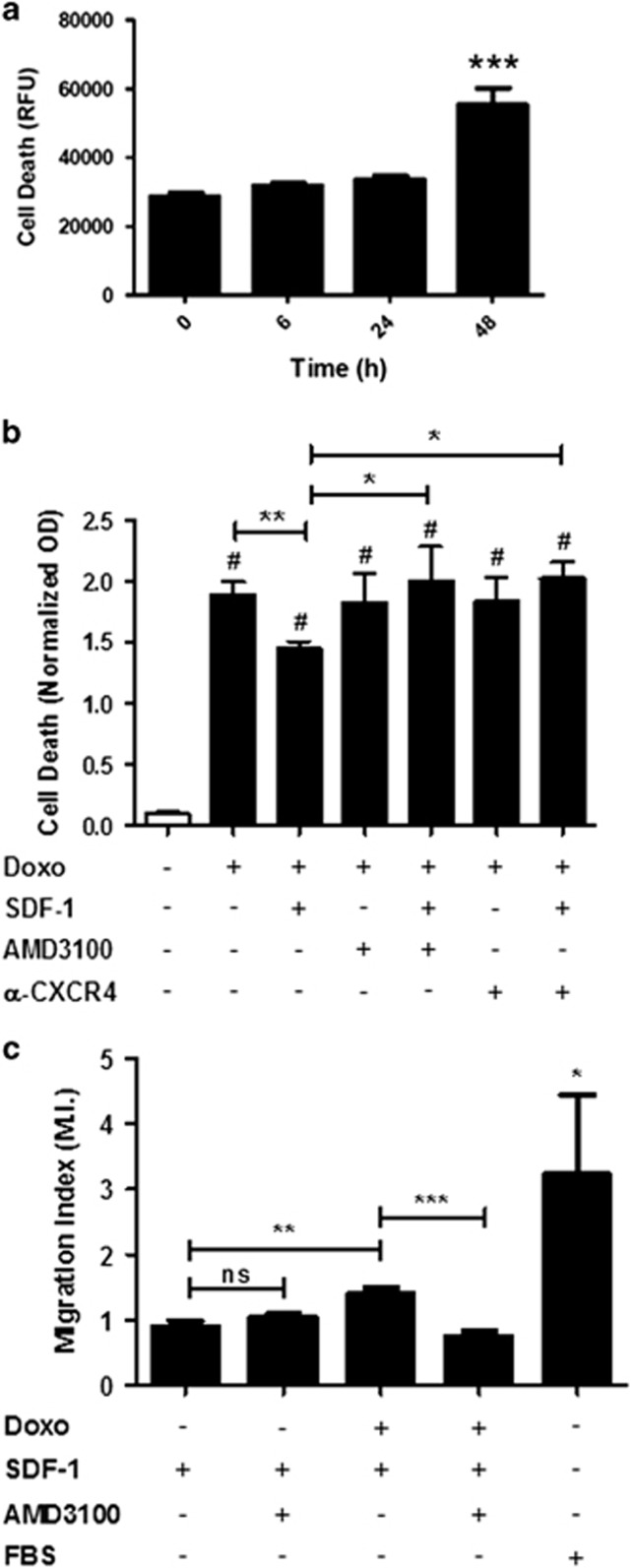
SDF1 protects and induces migration of DOXO treated CmPC. (**a**) Cell death analysis of CmPC upon DOXO treatment for increasing periods of time starting from 6 h up to 48 h as indicated. Cell death increased significantly 48 h after DOXO treatment (*n*=4, ***P<0.001). (**b**) Cell death analysis of CmPC exposed or not to 1 *μ*M DOXO. DOXO was added to CmPC for 24 h and then removed. SDF1, AMD3100 and *α*-CXCR4 were added after 24 h at the following dosages: SDF-1 at 100 ng/ml, AMD3100 at 3.2 *μ*g/ml and *α*-CXCR4 at 10 *μ*g/ml. Cell death was determined at 48 h. Mean values±SEM of three independent experiments run in triplicate (*P<0.05; ***P<0.0001). (**c**) Chemotatic responses of CmPC exposed or not to 1 *μ*M DOXO for 24 h in response to 100 ng/ml SDF1 or 10% FBS. Migration efficiency of CmPC was estimated using a Transwell assay. After 16 h of incubation transmigrated cells were quantified using crystal violet. The results are expressed as fold change of the untreated control cells exposed or not to DOXO. DOXO—cells treated with 1 *μ*M DOXO for 24 h; AMD3100—serum starved CmPC treated AMD3100 25 *μ*g/ml during migration; DOXO+AMD3100—cells treated with 1 *μ*M DOXO for 24 h and AMD3100 25 *μ*g/ml during migration (*n*=6, *P<0.05; **P<0.005; ***P<0.0001)

To assess the relationship and timing between DOXO-treatment and cell death, we performed a DOXO treatment time course followed by cell death analysis in CmPC (6, 24 and 48 h). We observed a significant induction of cell death after 48 h of DOXO treatment (Figure 3a). Successively, to address the role of SDF1 on cell survival, human CmPC were treated with DOXO for 24 h. Afterwards, DOXO was removed and human CmPC were kept for an additional 24 h in medium supplemented with SDF1 or medium alone. As expected, DOXO-induced cell death and SDF1 decreased the cell death index by 23%±9 (*P*<0.01). Importantly, the SDF1 protective effect was significantly reverted by the addition of the CXCR4-specific antagonist AMD3100 (*P*<0.05), as well as the specific CXCR4 blocking antibody^27^ (*P*<0.05; Figure 3b). To determine whether the SDF1/CXCR4 pathway was functional, human CmPC were treated or not with DOXO for 24 h and, successively, migration was stimulated with SDF1 for 16 h. Treatment with fetal bovine serum (FBS) was used as a positive experimental control. Intriguingly, we found that SDF1-mediated migration was significantly increased in CmPC treated with DOXO. As expected, SDF1-induced migration was significantly inhibited by the CXCR4 selective antagonist AMD3100^28^ only after DOXO treatment. Indeed, AMD3100 did not exert any effect upon SDF1 treatment since CmPC do not express CXCR4 in absence of DOXO (Figure 3c). This result demonstrated that human CmPC migration was specifically mediated by the activation of SDF1/CXCR4 axis (Figure 3c). Taken together, these results demonstrated that the SDF1/CXCR4 axis was functionally coupled to downstream signaling in CmPC.

### Doxorubicin modulates miR-200c/ZEB1 pathway in CmPC

Since DOXO cardiotoxicity has been ascribed to oxidative stress and DNA damage, we tested the possibility that DOXO could affect the expression of miR-200c and its target protein ZEB1, that our group demonstrated to be modulated by oxidative stress and to stimulate apoptosis and senescence of HUVECs *via* the upregulation of miR-200c and the downregulation of ZEB1.^24^

**Figure 4:**
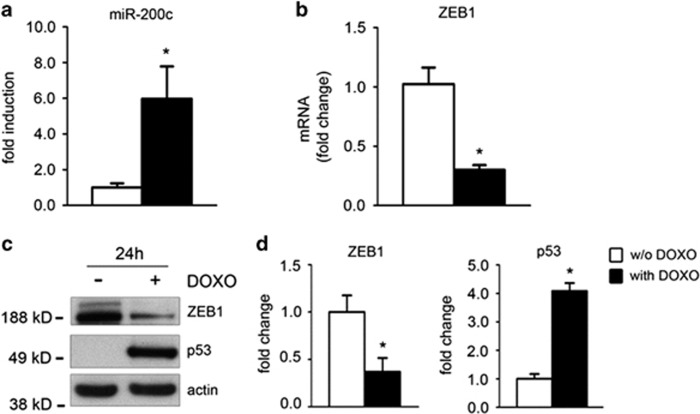
DOXO treatment upregulates miR-200c and downregulates ZEB1. (**a**) miR-200c expression upon 24 h DOXO treatment. miR-200c was significantly induced by DOXO treatment (*n*=4, *P<0.05). Results are presented as mean±S.E.M. (**b**) ZEB1 mRNA expression was quantified by qPCR; ZEB1 mRNA decreased upon DOXO treatment at 24 h (*n*=4, **P<0.01). Results are presented as mean±S.E.M. (**c**) CmPC were treated with 1 *μ*M DOXO for 24 h. ZEB1 and p53 proteins were evaluated by Western blot analysis. A representative western blot showing DOXO treatment induced p53 protein expression and ZEB1 downregulation. *β*-actin was used as a loading control. Actin indicates *β*-actin (**d**) Expression levels of ZEB1 and p53 protein were evaluated by densitometric analysis and normalized by *β*-actin protein levels (*n*=3, *P<0.05)

We found that miR-200c was upregulated after 24 h of DOXO treatment (Figure 4a). Conversely, the mRNA level of the miR-200c target ZEB1 was downregulated (Figure 4b).

We have previously demonstrated that p53 is necessary for miR-200c upregulation by oxidative stress in EC^24^ and DOXO treatment is known to induce p53 and oxidative stress;^29^ in keeping, we found that ZEB1 protein was downregulated 24 h after DOXO treatment, a time point at which p53 protein expression was upregulated (Figures 4c and d).

### ZEB1 inhibits CXCR4 expression

**Figure 5:**
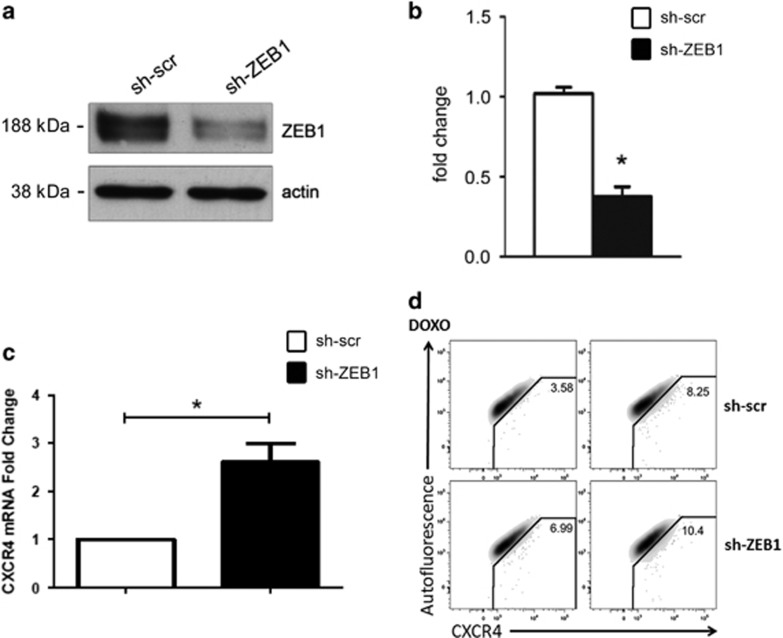
ZEB1 knockdown elicits CXCR4 increase. CmPC were infected either with the lentivirus carrying ZEB1-specific shRNA or with the control virus. After 24 h, cells were selected with puromycin. (**a**) Representative western blot demonstrating a 60% knockdown of ZEB1 expression in CmPC infected with a lentivirus encoding a ZEB1-specific shRNA sequence. (**b**) Expression levels of ZEB1 protein were evaluated by densitometric analysis and normalized by *β*-actin protein levels (*n*=3, *P<0.02). (**c**) CmPC transduced with a ZEB1-specific shRNA showed an upregulation of CXCR4 mRNA compared with control. (**d**) CmPC transduced with a ZEB1-specific shRNA displayed CXCR4 protein expression on the cell surface of CmPC, and increased both in absence of DOXO (upper and bottom left panels) and in presence of DOXO (upper and bottom right panels)

As a further confirmation of the inhibitory role of ZEB1 on CXCR4 expression, we knocked-down ZEB1 expression using a specific shRNA sequence (Figures 5a and b). We found that ZEB1 knockdown provoked CXCR4 mRNA upregulation (Figure 5c). Moreover, ZEB1 knockdown increased CXCR4 protein expression on the cell surface of CmPC both in the absence (Figure 5d upper and bottom left panels) and following DOXO exposure, when the DOXO-induced CXCR4 increase was further enhanced by ZEB1 depletion (Figure 5d upper and bottom right panels).

These data further confirm that ZEB1 represses CXCR4 expression in CmPC.

### ZEB1 binds to the promoter and the intronic region of the CXCR4 gene

ZEB1 is a transcription factor that binds to E-box sites which usually act as an inhibitor of target genes transcription.^30^ Therefore, we evaluated whether the CXCR4 gene could be a possible ZEB1 transcriptional target.

**Figure 6:**
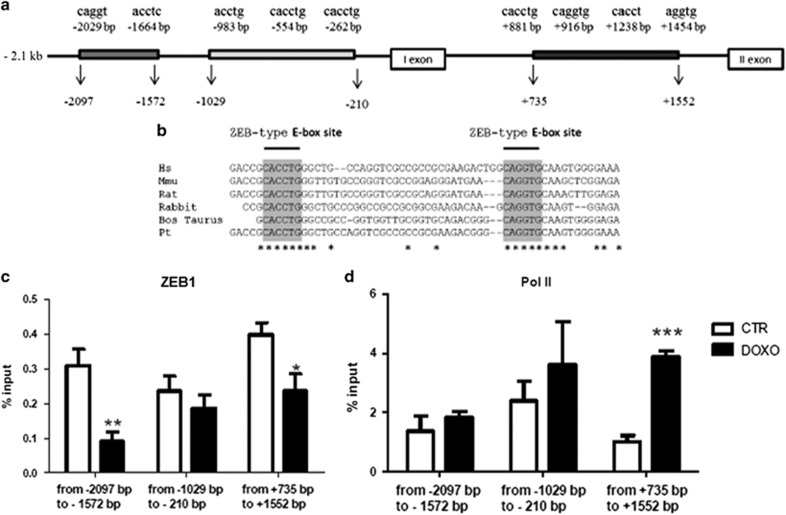
CXCR4 is a ZEB1 target gene. (**a**) Schematic figure showing the E-box binding sites of ZEB1 in the promoter and in the intronic region of the human CXCR4 gene. Five E-box sites are present at position – 2029 bp, −1644 bp, −983 bp, −554 bp, −262 bp upstream the first exon and four in the intronic region, at position +881 bp, +916 bp, +1238 bp, +1454 bp. (**b**) ZEB1 consensus sites revealed that the E-box sites at +881 bp and +916 bp are highly conserved across species of humans, mice, rats, rabbits, bos taurus and pan troglodytes. (**c**) ChIP assay of CmPC in the absence or presence of 1 *μ*M DOXO treatment for 24 h was performed with a ZEB1 antibody, followed by quantitative real-time PCR (ChIP-qPCR), using specific primers encompassing ZEB1 consensus sequences. (**d**) ChIP-qPCR CmPC in the absence or presence of 1 *μ*M DOXO treatment for 24 h was performed with a Pol II-specific antibody

Examination of 2.1 kbof CXCR4 human promoter and its intronic region revealed the existence of five E-box sites at positions −2029 bp, −1644 bp, −983 bp, −554 bp, −262 bp upstream of the first exon and four in the intronic region at positions +881 bp, +916 bp, +1238 bp, +1454 bp (Figure 6a). Interestingly, comparison of the human, mouse, rat, rabbit, bos taurus and pan troglodytes CXCR4 regions, encompassing the ZEB1 consensus sites, revealed that the E-boxes at +881 bp and +916 bp are highly conserved across species, suggesting the functional importance of these intronic sequences for CXCR4 expression (Figure 6b). Site-specific Chromatin immunoprecipitation (Chip) experiments followed by quantitative real-time PCR (ChIP-qPCR) indicated, although to different extents, a recruitment of ZEB1 and Pol II to the promoter region up to −2097 bp upstream of the first exon and in the intronic region encompassing from +735 to +1552 bp (Figures 6c and d).

In order to verify whether the increased expression of CXCR4 observed upon DOXO treatment was associated with a decrease of the binding activity of ZEB1 to the CXCR4 promoter, we performed ChIP experiments in cells treated with 1 *μ*M DOXO for 24 h. Results showed that ZEB1 occupancy was consistently reduced after DOXO treatment both in the promoter region encompassing the −2097 bp to −1572 bp upstream of the first exon and in the intronic E-boxes from +735 to +1552 bp. ZEB1 recruitment was still inhibited, but not significantly, in the promoter region encompassing −1029 bp to −210 bp (Figure 6c). Interestingly, Pol II binding to the proximal promoter region was increased after DOXO treatment both in the promoter sequence, although not significantly, and significantly in the intronic region, suggesting a higher CXCR4 promoter activity upon DOXO treatment (Figure 6d). These data indicate a role of ZEB1 as a transcriptional repressor of CXCR4 expression and its involvement in cellular response to DOXO treatment.

### SDF1 partially rescues cardiac dysfunction induced by doxorubicin

**Figure 7:**
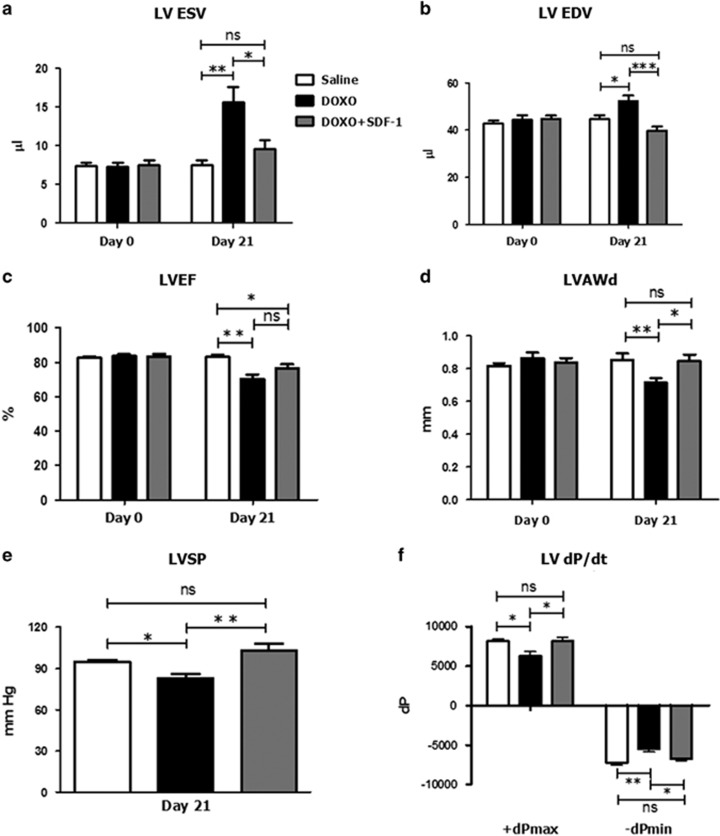
SDF1 partially rescues cardiac dysfunction induced by DOXO. Echocardiographic examination was performed on mice before (Day 0) and 21 days after the administration of DOXO (*n*=13), DOXO+SDF1 (*n*=13) or saline (*n*=13); (**a**) LV end-systolic volume (LVESV), (**b**) LV end-diastolic volume (LVEDV), (**c**) LV ejection fraction (LVEF), (**d**) LV anterior wall thickness at diastole (LVAWd). Evaluation of LV hemodynamic function with a Millar micro-tip catheter was performed at day 21; (**e**) LV systolic pressure (LVSP), (**f**) maximal rate of pressure development (+d*P*/d*t*) and maximal rate of pressure relaxation (-d*P*/d*t*). Saline, *n*=10; DOXO, *n*=10; DOXO+SDF1, *n*=13. Results are presented as mean±SD. (*P<0.05; **P<0.01; ***P<0.001)

The administration of SDF1 has been shown to promote cardioprotection in both animal models and in humans.^31^ Therefore, we examined whether SDF1 exhibited a cardioprotective action in DOXO-treated mice. SDF1 was administrated in a mouse model of DOXO-induced cardiotoxicity recently established in our laboratory^32^ (details are reported in the Materials and Methods Section and Supplementary Fig S3a). Left ventricular (LV) function was measured by transthoracic echocardiography before and 3 weeks after DOXO alone or DOXO+SDF1 or saline. No differences were found among groups before drug treatment (Figures 7a–d).

At day 21, LV dysfunction was greater in DOXO-treated mice with respect to the control group, as shown by a significant increase in LV end systolic volume (LVESV), LV end diastolic volume (LVEDV) and by a reduction in LV ejection fraction (LVEF) and LV anterior wall thickness at diastole (LVAWd; Figures 7a–d), in accordance with previous findings of our group.^32^

Notably, DOXO+SDF1 treatment reverted LV dysfunction at day 21, as evidenced by a significant decrease of both LVESV (Figure. 7a; *P*<0.05) and LVEDV (Figure 7b; *P*<0.001) and increase of LVAWd (Figure. 7d; *P*<0.05) *versus* DOXO-treated mice. In contrast, DOXO+SDF1-induced slight but not significant increase in LVEF (Figure 7c). Similar results were obtained by invasive measurements with a Millar catheter. As shown by a significant reduction of LV systolic pressure (LVSP; Figure 7e) and±d*P*/d*t* (Figure 7f), DOXO treatment impaired both LV contractility and relaxation with respect to controls. Accordingly, DOXO+SDF1 treatment significantly recovered these parameters with respect to DOXO-treated mice (Figures 7e and f).

### SDF1 partially rescues DOXO-dependent cardiac dysfunction via a miR-200c/ ZEB1/p53 pathway modulation

We successively asked whether SDF1 cardioprotection was due to the modulation of miR-200c/ZEB1/p53 pathway.

**Figure 8:**
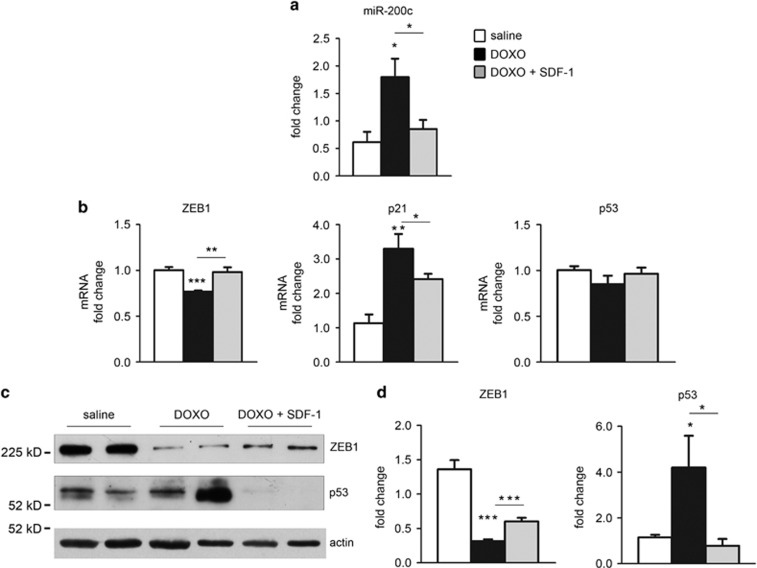
SDF1 partially rescues DOXO-dependent cardiac dysfunction via a miR-200c/ZEB1/p53 pathway modulation. Mice were treated with DOXO, DOXO+SDF1 or saline for 21 days. (**a**) Then mRNA was extracted from LV and analyzed for miR-200c, p53, ZEB1 and p21 mRNA expression levels. p53 mRNA was not modulated either by DOXO or DOXO+SDF1. miR-200c, and p21 mRNA were induced by DOXO and were all significantly decreased by SDF1 treatment. ZEB1 mRNA was downregulated by DOXO and returned to control levels by SDF1 treatment (Saline, *n*=5; DOXO, *n*=5; DOXO+SDF1, *n*=5; *P<0.05; **P<0.01; ***P<0.001). (**b**) Representative western blot with anti-p53 and ZEB1 antibodies showed that p53 upregulation by DOXO was decreased by SDF1 treatment and ZEB1 demise was reverted by SDF1 (Saline, *n*=5; DOXO, *n*=5; DOXO+SDF1, *n*=5). (**c**) Expression levels of p53 and ZEB1 protein were evaluated by densitometric analysis and normalized by *β*-actin protein levels (*n*=5; *P<0.05; ***P<0.001)

To this end, we evaluated miR-200c in LV heart extracts of mice treated with DOXO and we found that miR-200c expression levels were significantly increased compared to saline-treated mice (Figure 8a). Interestingly, SDF1 treatment upon DOXO significantly reduced miR-200c levels (Figure 8a). Moreover, we tested the mRNA expression levels of ZEB1 and we observed that, inversely to miR-200c, ZEB1 mRNA was downregulated by DOXO and returned to control levels in DOXO+SDF1-treated mice (Figure 8b). We confirmed by western blot analysis of the same specimens that ZEB1 protein, as well, is downregulated by DOXO and increased in DOXO+SDF1-treated hearts (Figures 8c and d).

We also tested p53 mRNA expression levels and we did not find a significant modulation upon DOXO or DOXO+SDF1 treatment (Figure 8b). On the other end p53 protein is induced by DOXO and decreased in DOXO+SDF1 treated extracts (Figures 8c and d).

In addition, we evaluated the mRNA of the CDK inhibitor p21Waf1/Cip1/Sdi1 (p21) expression levels, which is a direct transcriptional target of p53^33^ and ZEB1 (known to inhibit transcriptionally p21).^34^ In keeping, we found an upregulation of p21 mRNA in DOXO treated mice and a significant decrease in DOXO+SDF1 treated hearts (Figure 8b).

## Discussion

The cardioprotective role of the SDF1/CXCR4 axis was already established in myocardial infarction,^35, 36, 37, 38^ however, it was never studied in DOXO-induced cardiotoxicity.

The effect of DOXO administration has been mainly focused on its toxic effects on breast cancer cells. However, according to the most recent literature,^11, 13^ our results indicated that DOXO treatment markedly affects the pool of cardiac resident progenitors, including CmPC. At the molecular level, we observed that DOXO induces the upregulation of CXCR4, making human CmPC more prone to respond to SDF1 stimulation. Indeed, we demonstrated that the administration of SDF1 is able to protect CmPC from DOXO-induced cell death and to induce their migration, demonstrating that the SDF1/CXCR4 axis is functionally coupled to downstream signaling.

Moreover, we demonstrated a novel implication of miR-200c/ZEB1 pathway in response to DOXO. We have previously showed that p53 is necessary for miR-200c upregulation by oxidative stress in EC.^24^ Since DOXO is known to induce p53 and oxidative stress,^29^ the upregulation of miR-200c was expected.

Further, our results proved that miR-200c upregulation and the concomitant inhibition of its target ZEB1 is implicated in the increased expression of CXCR4.

As a matter of fact, ZEB1 is a transcriptional inhibitor and we here showed that ZEB1 binds both to the promoter and to the intronic sequence (the region upstream of the first exon), inhibiting CXCR4 transcription.

This intronic sequence has been demonstrated to play an important regulatory role for CXCR4 expression in different papers,^39, 40^ and for this reason we tested also this gene sequence for ZEB1 binding. Moreover, in this region we found two E-boxes highly conserved across species, suggesting the functional importance of these intronic sequences for CXCR4 regulation.

Interestingly, upon DOXO treatment, ZEB1 binding on the CXCR4 promoter and intronic sequence decreased and Pol II binding to the proximal promoter region increased both in the promoter sequence and in the intronic region, suggesting a higher CXCR4 promoter activity upon DOXO treatment.

In keeping with this, the upregulation of CXCR4 by the miR-200 family was already reported in mouse embryonic stem cells upon nitric oxide treatment.^41^ In this study the mechanism of CXCR4 upregulation involved another transcription factor targeted by the miR-200 family that is Zinc-Finger-E-Box-Binding-Homeobox-2 (ZEB2), which also binds to and inhibits E-boxes.^41^

Notwithstanding acute upregulation of miR-200c ultimately leads to CXCR4 upregulation and consequent SDF1-induced amelioration of cardiotoxicity, chronic upregulation of miR-200c might be one of the leading causes for the establishment of DOXO-induced apoptosis and senescence. In fact, we previously demonstrated that oxidative stress inducing miR-200c causes ZEB1 downregulation which enhances apoptosis and senescence in EC and in different cell types.^24^ Recently, we demonstrated that miR-200c upregulation is responsible of reactive oxygen species production and nitric oxide decrease by targeting three important proteins involved in endothelial function (i.e., Sirtuin1, endothelial nitric oxide synthase and Forkhead box O1), further supporting a role for this miRNA in cardiotoxicity.^42^

Notably, CXCR4 upregulation preceded DOXO-induced cell death, therefore, if the time frame of SDF1 administration is precocious, it can prevent the negative effects caused by anthracyclines.

In the present study, we demonstrated for the first time, that *in vivo* administration of SDF1 attenuates DOXO-induced LV remodeling and functional impairment producing a significant recovery of LVSP and±dP/dt reduction when compared to DOXO-treated mice.

Moreover, our results indicated that in LV specimens of mice treated with SDF1+DOXO there is a decrease of miR-200c and p53 upregulation caused by DOXO and a restoration of ZEB1 expression, explaining SDF1 positive effect also via the modulation of this molecular pathway. In keeping, p21 mRNA, that is induced by p53^33^ and inhibited by ZEB1,^34^ is induced by DOXO treatment and is decreased by SDF1 administration (Supplementary Fig. S4).

Cumulatively, our study provided the first evidence that: (1) DOXO promotes a compensatory response in human CmPC by the CXCR4 upregulation, making this cardiac mesenchymal subpopulation more prone to respond to SDF1 protective stimuli; (2) DOXO induces the upregulation of miR-200c which, in turn, downregulates ZEB1; 3) ZEB1 binds to the CXCR4 promoter repressing its expression (Supplementary Fig. S4); (4) the activation of the SDF1/CXCR4 axis is protective both *in vivo* and *in vitro* against the adverse cardiac events induced by DOXO; (5) SDF1 treatment is able to rescue cardiac dysfunction via a miR-200c/ ZEB1/p53 pathway modulation.

Our study revealed new possible molecular mechanisms associated with DOXO treatment that could allow the improvement of current therapeutic approaches or the development of novel strategies and hopefully lead to the increase in the number of long-term survivors experiencing anticancer therapy cardiotoxicity.

## Materials and Methods

### *Ex-vivo* immunofluorescence and confocal analysis

Experiments were performed in accordance with the national and international law and policies (4D.L. N.116, G.U., supplement 40, 18-2-1992; EEC Council Directive 86/609, OJ L 358,1,12-12-1987) and the guidelines indicated in the Declaration of Helsinki. The protocol was approved by the University Committee on Animal Resources at the University of Milan. All efforts were made to minimize animal suffering.

Female C57Bl/6 wild-type mice (CharlesRiver Laboratories, Italy) aged 8 to 10 weeks were randomly divided into two groups.^32^ The DOXO group (*n*=7) received six equal intraperitoneal DOXO injections over a period of 2 weeks (4 mg/kg each; cumulative dose, 24 mg/kg). The control group (*n*=7) was treated with physiological saline in the same manner as the regimens for the DOXO group.

After imaging, mice were killed and weighed, then hearts were blocked in diastole by intracardiac injection of 100 ml potassium chloride (3 mol/l). Mouse hearts were fixed in 10% formalin and embedded in paraffin. For immunofluorescence analysis, sections were deparaffinized, rehydrated, and boiled for 20 min in an antigen retrieval buffer (sodium citrate, pH=6.0, DAKO). Following antigen retrieval, sections were incubated with primary antibody at 4 °C overnight. The following primary antibodies were used: anti-CXCR4 (Abcam, Cambridge, UK), anti-CD44 (Abcam), anti-*α*-sarcomeric actin (Sigma-Aldrich, Milan, Italy), and anti-CD29 (Abcam). After washing, sections were incubated with the following fluorochrome-conjugated antibodies for 1 h at room temperature in the dark: Alexa488 and Alexa546 (Invitrogen, Milan, Italy). Nuclear staining was performed by incubating sections with Hoechst 33342 (Sigma-Aldrich). Sections were observed using a Zeiss LSM 710 confocal microscope (Zeiss, Milan, Italy). Images were acquired at × 40 magnification to count the CXCR4 or the double-positive cells (CXCR4/CD44 and CXCR4/CD29). The CXCR4^+^ cells were calculated as a percentage of the total number of nuclei in the same fields (nine different fields per mouse). CXCR4/CD44^+^ cells were calculated as a percentage of the total number of CD44^+^ cells in the same fields (three different fields per mouse, data not shown).

### CmPC isolation and culture

Right auricles were obtained from donors undergoing cardiac surgery after Local Ethics Committee approval (protocol CCFM C9/607) and signed informed consent in accordance with the Declaration of Helsinki. CmPC were obtained by adapting two different methods previously described.^43, 44^

### Western blot

Whole cell lysates were obtained by harvesting cells with Laemmli buffer, containing 100 mM Tris (pH 6.8), 20% glycerol, 4% SDS. Protein concentration was determined by BCA protein assay kit (Pierce) following the manufacturer's instructions. Then, DTT (200 mM) was added and lysates were boiled for 5 min. Proteins were separated in SDS polyacrylamide gels and transferred to nitrocellulose by standard procedures. The following antibodies were used to detect the proteins of interest: anti-CXCR4 (Ab-2074; Abcam, UK); anti-ZEB1 (H-102; Santa Cruz Biotechnology, Heidelberg, Germany); anti- human p53 (Ab-6, DO-1 Oncogene Research Products); anti-murine p53 (Ab-1; Oncogene Research Products, Boston, MA, USA); anti-actin (AC15; Sigma).

### Quantitative reverse transcriptase–polymerase chain reaction

Total RNA was purified from CmPC at passage 4 (P4) using Trizol reagent (Invitrogen) according to the manufacturer’s instructions. miR-200c expression was evaluated by quantitative reverse transcriptase-PCR (qRT-PCR) using single TaqMan microRNA assays (Life Technologies, Milan, Italy), according to manufacturer’s instructions. Retro-transcription of 5 ng of total RNA was conducted using the TaqMan MicroRNA Reverse Transcription Kit (Life Technologies, Milan, Italy), followed by amplification with specific primers on a 7900HT Fast Real-Time PCR System (Life Technologies). Results were expressed as cycle threshold (Ct) levels and normalized using miR-16 as a calibrator. mRNAs levels were analysed using the SYBR-GREEN qPCR method (5 ng per assay, Qiagen) and quantified with ABI Prism 7000 SDS (Applied Biosystems). Relative expression was calculated using the comparative Ct method (2^–ΔΔCt^). mRNA expression was normalized for Beta-2-microglobulin (B2M) levels.

The following primers were used for SYBR-GREEN Real-Time PCR:

humanZEB1:

forward:5′-GGGAGGAGCAGTGAAAGAGA-3′

reverse: 5′-TTTCTTGCCCTTCCTTTCTG-3′

humanB2M:

forward:5′-TTCTGGCCTGGAGGCTATC-3′

reverse:5′-TCAGGAAATTTGACTTTCCATTC-3′

human CXCR4:

forward: 5′-TTGTCATCACGCTTCCCTTCT-3′

reverse: 5′-CATGGACTGCCTTGCATAGGA -3′

murine ZEB1:

forward: 5′-AGGTGATCCAGCCAAACG-3′

reverse: 5′-GGTGGCGTGGAGTCAGAG-3′

murine TP53:

forward: 5′-CAGTCTGGGACAGCCAAGTC-3′

reverse: 5′-CAGCTGGCAGAATAGCTTATTGA-3′

murine p21:

forward: 5′-TCCACAGCGATATCCAGACA-3′

reverse: 5′-GGACATCACAGGATTGGAC-3′

murine RPL13:

forward: 5′- CTCGGCCGTTCTCCTGTAT-3′

reverse: 5′- GTGGAAGTGGGGCTTCAGTA-3′

### Cell death quantification

The CellTox Green Cytotoxicity Assay (Promega, Milan, Italy) was used to test cell death in CmPC cells treated with 1 *μ*M DOXO for increasing period of times (6, 24, 48 h, Figure 3a). This assay measures changes in membrane integrity that occur as a result of cell death, using a dye that is excluded from viable cells but preferentially stains the DNA from dead cells. When the dye binds DNA released from cells, its fluorescence properties are substantially enhanced. Therefore, the fluorescence signal produced by the interaction with DNA from dead cells is proportional to cytotoxicity.

Apoptosis/necrosis in CmPC migration conditions (Figure 3b) were assessed by using a cell death detection ELISA kit (Roche Diagnostics, Basel, Switzerland). Quantification of histone-complexed DNA fragments (mono- and oligonucleosomes) was performed by one-step sandwich immunoassay, measuring nucleosome-bound DNA fragments by photometric analysis.

### Migration assay

CmPC migration assay was evaluated in a 24-well modified Boyden chamber as previously described.^45^ Briefly, CmPC were seeded in the upper chamber of a modified Boyden chamber (Corning Corporation; 8-mm pore size) and exposed or not (CTR) to DOXO 1 *μ*M for 24 h under static conditions in Ham’s F12 medium. The upper chamber was then placed in a 24-well culture dish containing different stimuli: 500 *μ*l of Ham’s F12 medium supplemented with 100 ng/ml of SDF1 (R&D Systems) or 10% FBS (positive control) or Ham’s F12 medium alone (negative control). After 16 h of incubation at 37 ^o^C, 5% CO_2_, transmigrated cells were counted. Non-migratory cells on the upper side of the membrane were scraped off with wet cotton swabs. Cells present both in the lower chamber and on the lower side of the filter were counted and considered as migrated cells. Each well was washed with PBS with Ca^2+^/Mg^2+^ (Gibco, Life Technologies, Milan, Italy) to remove debris and floating cells, then adherent cells were fixed for 15 min with 4% paraformaldehyde (PFA) and washed 3 times. Cells were stained with 0.05% Crystal Violet (Sigma-Aldrich) for 5 min and washed 3 times with PBS. To quantify the cell number, the dye was solubilized with 10% acetic acid solution and the OD of each well was quantified at 540 nm with Mithras LB 940 (Berthold Technologies, Milan, Italy). The migration index (M.I.) was calculated by dividing the number of cells migrated in presence of SDF1 or FBS by the number of cells migrated in Ham’s F12 medium alone.

### Lentiviral infection

Lentiviral supernatants were produced using standard procedures. Briefly, HUVEC were infected for 2 h with lentiviral supernatants and then were allowed to recover in complete fresh medium for additional 24 h. Afterwards, puromycin-containing medium (0.5 *μ*g/ml, Sigma) was added to the cells. MISSION shRNA lentiviral control and ZEB1-specific constructs were purchased from Sigma. ZEB1 shRNA sequences were already tested for efficacy^24^ and used in this paper were: shRNAZEB1:

5′-CCGGGCTGTTGTTCTGCCAACAGTTCTCGAGAACTGTTGGCAGAACAACAGCTTTTT-3′.

### Flow cytometry

CmPC cultured in the indicated conditions were detached using a non-enzymatic method, cells were resuspended in PBS containing 0.1% BSA (Gibco) and 2 mM EDTA (Gibco) and incubated in the dark for 15 min with suitable combinations of the monoclonal antibodies or isotype-matched control monoclonal antibodies: CD29-PE, CD44-PE, CD73-PE, CD166-PE, CXCR4 APC (BD Pharmingen). Samples were then washed with 1 ml of washing buffer and centrifuged for 10 min at 400 × g at 4 °C to remove unbound antibodies. Cells were resuspended in 250 *μ*l of washing buffer and analyzed.

For Figure 5c, CmPC were incubated with Brilliant Violet 421(BV421)-conjugated anti-CXCR4 antibody (Biolegend, San Diego CA) used at 1 *μ*l in 30 *μ*l final dilution for 15 min at RT. The CXCR4 signal (450 nm wavelenght) was plotted against an empty channel of the FACSAria cell sorter (Beckton Dickinson, Franklin Lakes, NJ, USA) receiving autofluorescence detected at 530 nm wavelenght (AF 530) to set up proper positivity gates. Numbers indicate percentages of CXCR4-positive cells for the indicated conditions. Flow cytometry data was analyzed by FlowJo software ver 9.9.5 (Treestar, Ashland, OR, USA).

### Chromatin immunoprecipitation

Cells were incubated in 1% of formaldehyde for 10 min at 22 °C. The reaction was stopped by addition of glycine to a final concentration of 0.125 M. Chromatin immunoprecipitations were performed as previously described.^46^ Recovered DNA was analyzed by qRT-PCR. The following primer sequences were used: from −2097 bp to −1997 bp forward, 5′-TGCCAAATCCTACCTTCTTCTG-3′ and reverse, 5′-CTTCCTTCGGAGGATGTAGC-3′ from −1680 bp to −1572 bp forward, 5′-TTCCATCCACTTTAGCAAGGA-3′ and reverse, 5′-CTCCCAGAGGCATTTCCTAA-3′ from -1029 bp to -910 bp forward, 5′-GGTCCTGCAGTTCGAGAGTT-3′ and reverse, 5′-CCAGGTGCGGTCTTAACC-3′ from -324 bp to -210 bp forward, 5′-TGGCGTGGGTGTAGTGGG-3′ and reverse, 5′-TGATCCAGTTAACCCGGC-3′ from +735 bp to +871 bp forward, 5′-CACGAGGATGGCAAGAGAC-3′ and reverse, 5′-ACTTGTAGTGGGTAAAGAGAATGC-3′ from +1402 bp to +1552 bp forward, 5′-TGCAAACCATTTTGCTCCGA-3′ and reverse, 5′-AAACTCCTCCCTGCACGATG-3′. We used a ChIP assay to examine ZEB1 and Pol II occupancy at these sites, followed by quantitative real-time PCR (ChIP-qPCR). Specific primers were used to amplify DNA regions encompassing ZEB1 consensus sequences. To normalize ChIP-quantitative PCR data were analyzed with the Percent Input Method. Percent input was calculated by 100 × 2(^Ct^_Input_−^Ct^_Enriched_). No antibody values were subtracted.

### Animal grouping and SDF1 administration

Experiments were performed in accordance with national and international laws and policies (4D.L. N.116, G.U., supplement 40, 18-2-1992; EEC Council Directive 86/609, OJ L 358,1,12-12-1987) and the guidelines indicated in the Declaration of Helsinki. The protocol was approved by the University Committee on Animal Resources at the University of Milan. All efforts were made to minimize animal suffering.

Preliminary tests were performed to evaluate the *in vivo* effect of SDF1 administration. First, based on the data available in the literature, two concentrations of SDF1 (10 *μ*g/kg and 40 *μ*g/kg) were tested, identifying 10 *μ*g/kg as the minimum effective dose for the recruitment of CXCR4-positive cells from the bone marrow (data not shown).

Female C57Bl/6 wild-type mice (Charles River Laboratories) aged 8 to 10 weeks were randomly divided into three groups (Supplementary Fig. S3a). In the first group (DOXO, *n*=13), DOXO was administered in six equal intraperitoneal injections over a period of 2 weeks (4 mg/kg each; cumulative dose, 24 mg/kg). In the second group (DOXO+SDF1, *n*=13), DOXO was administrated as in the first group and SDF1 was administered in three equal intraperitoneal injections every 72 h, starting from the second week of DOXO treatment (10 *μ*g/kg each). In the third group (saline, *n*=13), control mice were treated with physiological saline in same manner as the regimens for the DOXO group. Then, the percentage of bone marrow-mobilized CXCR4^+^ cells following the systemic administration of SDF1 at a concentration of 10 *μ*g/kg was evaluated at different time points (6, 48 and 96 h). Results showed that the effect of SDF1 administration on CXCR4^+^ cell mobilization was maintained 48 h after treatment and was almost entirely absent at 96 h (Supplementary Fig. S3b). In order to maximize the effect with the least possible number of injections, we decided to treat mice with SDF1 every 72 h.

### Echocardiography

Cardiac ultrasound studies were performed prior to and 3 weeks after treatment using the Vevo 2100 high-resolution imaging system (VisualSonics) and a 40-MHz linear transducer with simultaneous electrocardiographic recording. Analyses were performed on mice lightly anesthetized with 0.5 to 1% isoflurane (heart rate, 480–550 beats/min), 1 day before starting treatment (baseline) and 21 days after drug administration (Figure 7). The anterior chest wall was shaved, acoustic coupling gel was applied, and the transducer was placed avoiding excessive pressure. Two-dimensional short-axis M-mode echocardiography was performed at the level of the midpapillary muscle to measure LVESV and LVEDV, LVAWd and LVEF.

### Hemodynamics

LV performance was analyzed using a Millar pressure-volume conductance catheter (SPR-839; Millar Instruments), as previously described.^32^ Briefly, at day 21, mice were anesthetized, the trachea was cannulated, and the animal was connected to a positive-pressure volume-controlled rodent ventilator (MiniVent). To measure the LVSP and maximal rate of pressure development (+dP/dt) and maximal rate of pressure relation (-dP/dt), the catheter was introduced through the right carotid artery into the ascending aorta and then into the LV cavity.
