# Supplementary material for: Doxorubicin upregulates CXCR4 via miR-200c/ZEB1-dependent mechanism in human cardiac mesenchymal progenitor cells
Source: Cell Death Dis. 2017 Aug 24;8(8):e3020–. doi: 10.1038/cddis.2017.409 (PMC5596590; doi:10.1038/cddis.2017.409)
Supplement: Supplementary Information [file cddis2017409x1.pdf]

Cell Death and Disease

Doxorubicin up-regulates CXCR4 *via* miR200c/ZEB1-dependent mechanism in human cardiac mesenchymal progenitor cells

Sara Beji[1]*, Giuseppina Milano[2,3]*, Alessandro Scopece[2], Lucia Cicchillitti[4], Chiara Cencioni[5,6], Mario Picozza[1], Yuri D'Alessandra[7], Sarah Pizzolato[2], Matteo Bertolotti[8], Gabriella Spaltro[2], Angela Raucci[8], Giulia Piaggio[4], Giulio Pompilio[2,9], Maurizio C. Capogrossi[1], Daniele Avitabile[2], Alessandra Magenta[1#*] and Elisa Gambini[2#*]

[1] Vascular Pathology Laboratory, Istituto Dermopatico dell'Immacolata, IRCCS, Via dei Monti di Creta 104, 00167 Rome, Italy
[2] Unit of Vascular Biology and Regenerative Medicine, Centro Cardiologico Monzino, IRCCS, Via Carlo Parea 4, 20138 Milan, Italy
[3] Laboratory of Cardiovascular Research, Department of Surgery and Anesthesiology, University Hospital Lausanne; Rue du Bugnon 46 1011 Lausanne, Switzerland
[4] Department of Research, Advanced Diagnostics and Technological Innovation, Regina Elena National Cancer Institute, Via Elio Chianesi 53, 00144 Rome, Italy
[5] Division of Cardiovascular Epigenetics, Department of Cardiology, Goethe University, Theodor-Stern-Kai 7, 60590, Frankfurt am Main, Germany
[6] National Research Council (CNR), Institute of Cell Biology and Neurobiology, Via del Fosso di Fiorano, 64, 00143 Rome, Italy
[7] Immunology and Functional Genomics Unit, Centro Cardiologico Monzino (CCM), IRCCS, Via Carlo Parea 4, 20138 Milan, Italy
[8] Unit of Experimental Cardio-oncology and Cardiovascular Aging, Centro Cardiologico Monzino (CCM), IRCCS, Via Carlo Parea 4, 20138 Milan, Italy
[9] Department of Clinical Sciences and Community Health, University of Milan, Via Festa del Perdono 7, 20122 Milan, Italy

* Equally contributed
#Co-Corresponding authors:
Elisa Gambini Tel: +39 02 5800 2027, Fax: +390258002342; email: elisa.gambini@ccfm.it ORCID:orcid.org/0000-0002-8859-8259
Alessandra Magenta Tel: +390689996615, Fax:+390666464456 email: ale.magenta@gmail.com ORCID: orcid.org/0000-0002-9054-337X

Supplementary Materials

Supplementary Figure Legends

Figure S1: CXCR4 expression *in vivo* in response to DOXO. a) Confocal analysis of CXCR4 and α-sarcomeric actin$^+$ cells in heart mice tissue. Representative images of heart tissue sections from DOXO and untreated mice immunostained with CXCR4 and α-Sarcomeric Actin (α-Sarc) antibody. Nuclei are stained with Hoechst 33258. Scale bar 20μm. **b)** Representative images of heart sections of treated and untreated mice with DOXO, immunostained with CXCR4 (green) and CD29 (red) antibody. Nuclei are stained with Hoechst 33258 (blue). Scale bar: 10μm and 20μm in the inset.

Figure S2: CXCR4 is expressed in CmPC in vitro and increased in response to Doxorubicin. Example of flow cytometric dot plot before and after treatment with DOXO 1μM for 24h, for surface CD29, CD44, CD73, CD166 and CXCR4 markers at P4. CmPC fully express CD44, CD29, CD73 and CD166 surface markers, confirming their mesenchymal cell origin. CmPC displayed CXCR4 protein expression on the cell surface of CmPC (1,36 ± 0,28%), and increase after DOXO treatment (9,54 ± 1,97%) (upper right panels).

Figure S3: *In vivo* experimental settings. a) *In vivo* Experimental design**.** C57Bl/6 wild-type mice were randomly divided into three groups. In the first group (DOXO, n = 10), DOXO was administered in six equal intraperitoneal injections over a period of 2 weeks. In the second group (DOXO+SDF1, n = 10), DOXO was administrated as in the first group and SDF1 was administered in three equal intraperitoneal injections every 72h, starting from the second week of DOXO treatment. In the third group (saline, n = 10), control mice were treated with physiological saline in the same manner as the regimens for the DOXO group. **b)** FACS analysis showing CXCR4$^+$ cells mobilized from the bone marrow at 0, 6, 48 and 96h of SDF1 administration. CXCR4 positive cells mobilization is maintained 48h after treatment and is almost entirely absent in 96h (*p<0.05; **p<0.005).

Figure S4: Proposed mechanism of DOXO mediated CXCR4 up-regulation and SDF1 role in cardioprotection. Doxorubicin induces DNA damage response, apoptosis and senescence, that are known to up-regulate p53, which enhances miR-200c expression. Thus, DOXO treatment results in miR-200c up-regulation and consequent down-regulation of its target, ZEB1. In keeping, p21 mRNA, that is induced by p53 and inhibited by ZEB1, is up-regulated by DOXO treatment. ZEB1 acts as a transcriptional inhibitor of CXCR4 by binding to the E-boxes sequences in the promoter and in the intronic region of CXCR4 gene. Therefore, DOXO-mediated ZEB1 down-regulation results in the increased expression of CXCR4. As a consequence, CXCR4 up-regulation in CmPC makes them more responsive to

SDF1. Activation of SDF1/CXCR4 pathway results in increased protection against DOXO, by the activation of pro-survival and migration pathways, that inhibit p53 up-regulation and consequently miR-200c and p21 transcription**.**

Supplementary Figures

Figure S1

a

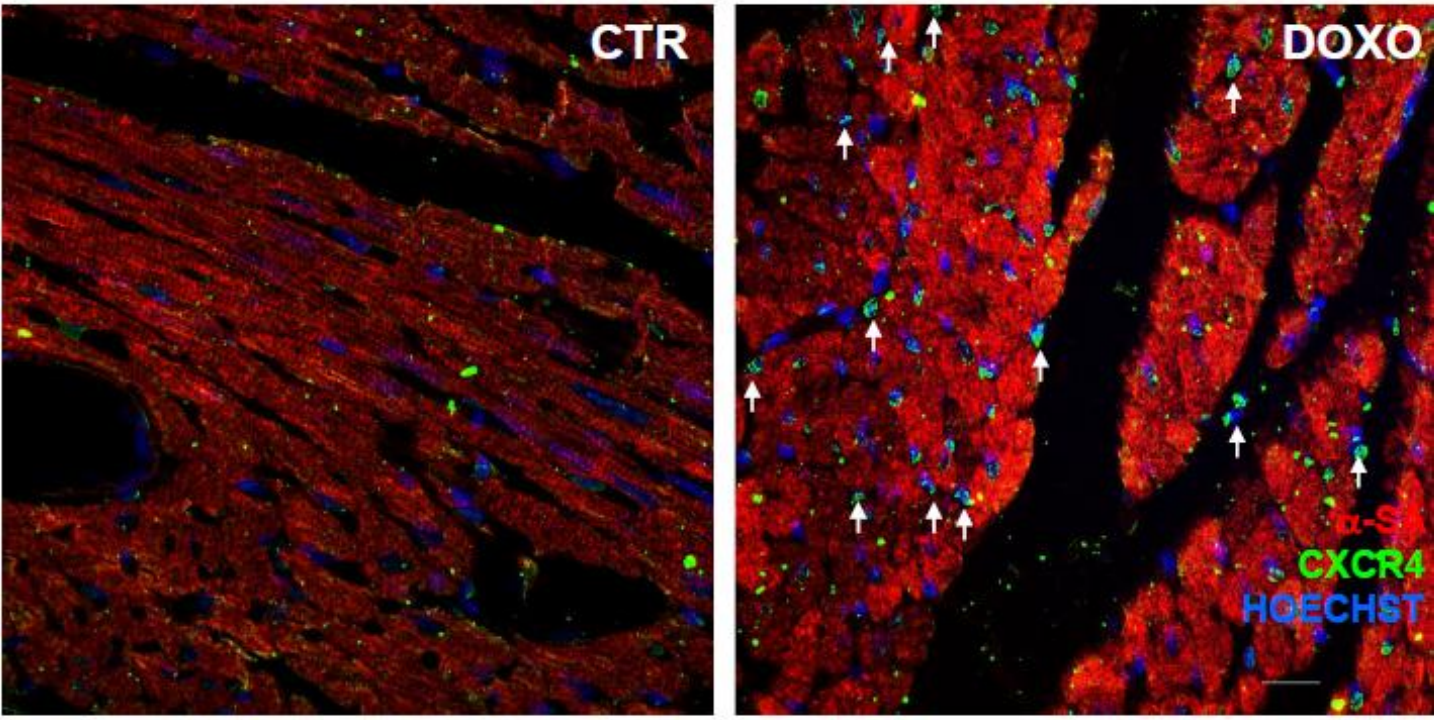

b

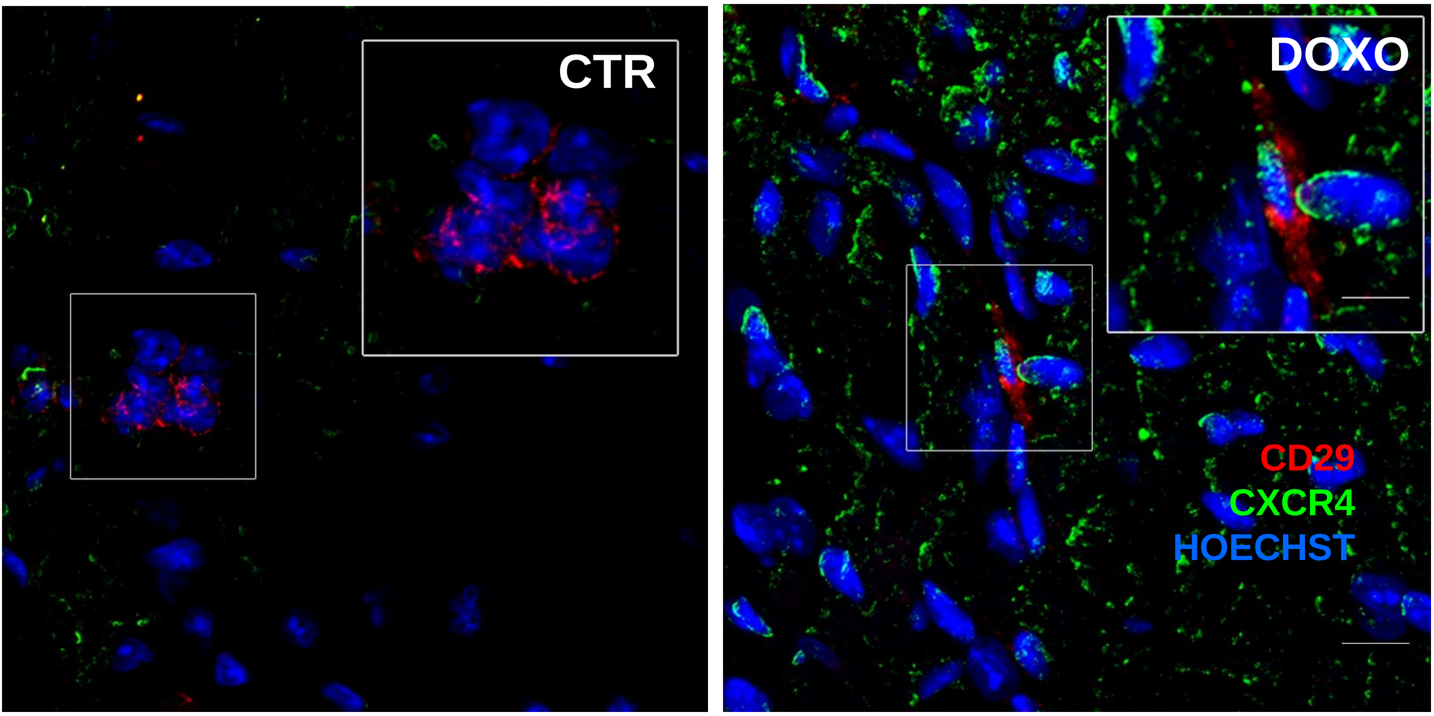

Figure S2

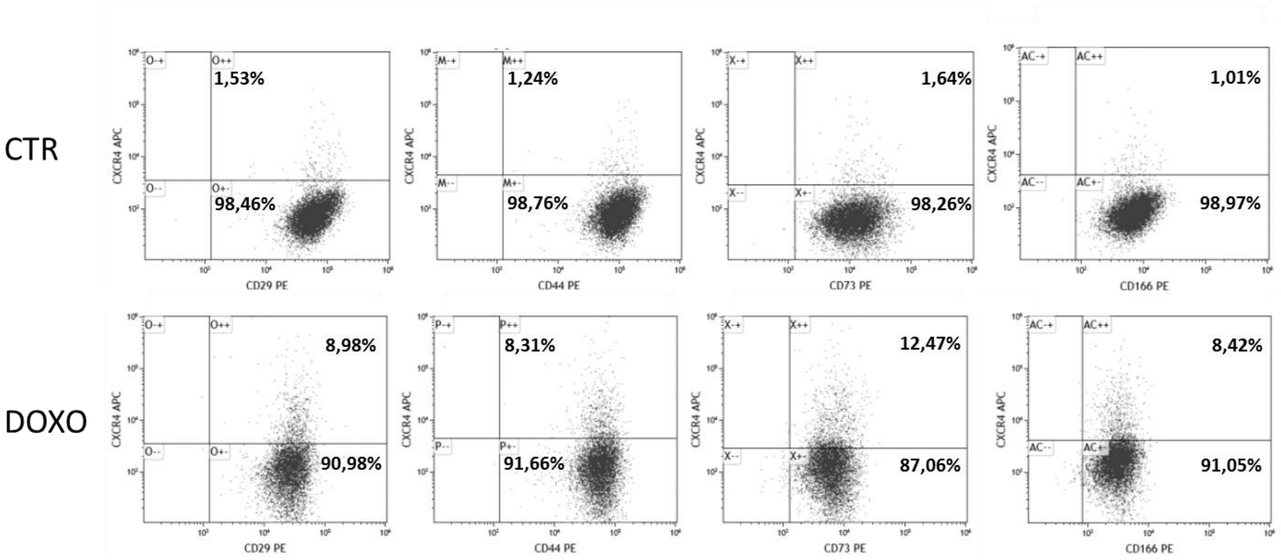

CTR
DOXO
CXCR4 APC
CD29 PE
CD44 PE
CD73 PE
CD166 PE
1,53%
98,46%
1,24%
98,76%
1,64%
98,26%
1,01%
98,97%
8,98%
90,98%
8,31%
91,66%
12,47%
87,06%
8,42%
91,05%

Figure S3

a

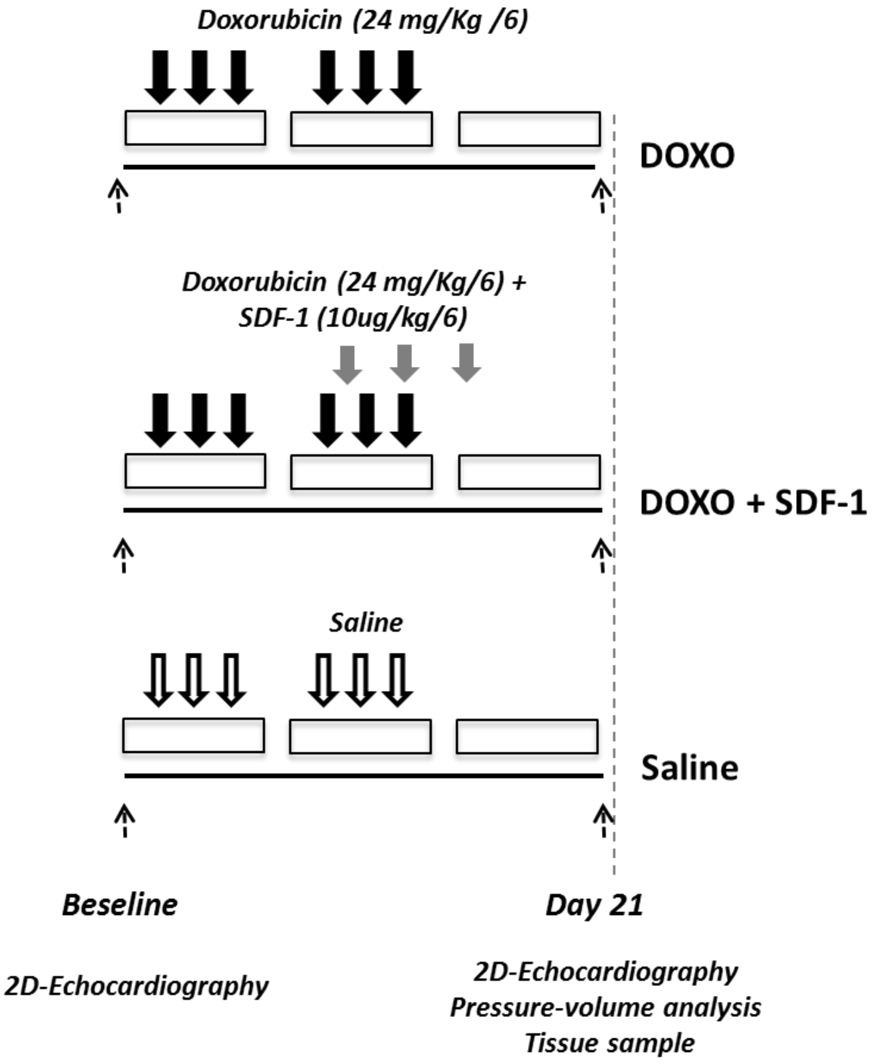

Doxorubicin (24 mg/Kg /6)
DOXO
Doxorubicin (24 mg/Kg/6) +
SDF-1 (10ug/kg/6)
DOXO + SDF-1
Saline
Saline
Beseline
Day 21
2D-Echocardiography
2D-Echocardiography
Pressure-volume analysis
Tissue sample

b

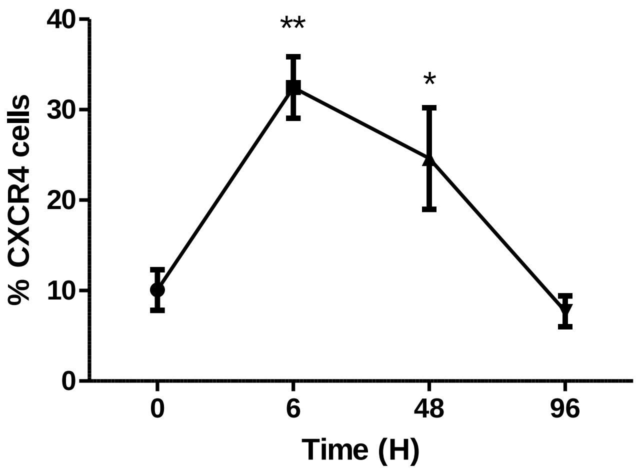

40
30
20
10
0
% CXCR4 cells
**
*
0
6
48
96
Time (H)

Figure S4

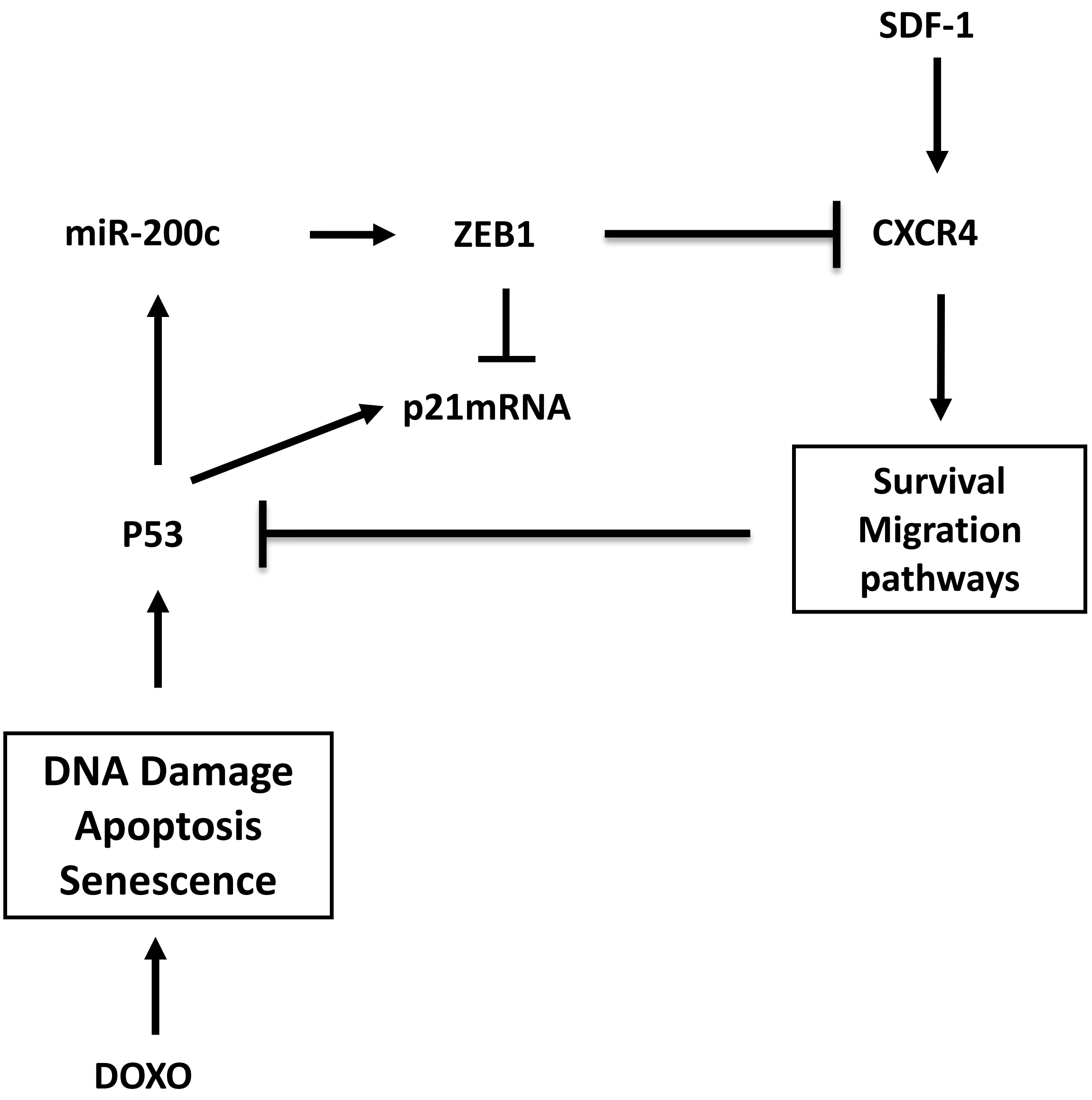
SDF-1
miR-200c
ZEB1
CXCR4
p21mRNA
P53
Survival
Migration
pathways
DNA Damage
Apoptosis
Senescence
DOXO
